# *Hugan Qingzhi* Exerts Anti-Inflammatory Effects in a Rat Model of Nonalcoholic Fatty Liver Disease

**DOI:** 10.1155/2015/810369

**Published:** 2015-06-14

**Authors:** WaiJiao Tang, Lu Zeng, JinJin Yin, YuFa Yao, LiJuan Feng, XiaoRui Yao, XiaoMin Sun, BenJie Zhou

**Affiliations:** ^1^Center for Drug Research and Development, Zhujiang Hospital, Southern Medical University, Guangdong, Guangzhou 510282, China; ^2^Department of Pharmacy, Ganzhou People's Hospital, Nanchang University, Jiangxi, Ganzhou 341000, China; ^3^Department of Pharmacy, The Third Affiliated Hospital of Guangzhou Medical University, Guangzhou 510150, China; ^4^Department of Pharmacy, Beijiao Hospital, Southern Medical University, Guangdong, Guangzhou 528311, China; ^5^Department of Traditional Chinese Medicine, Zhujiang Hospital, Southern Medical University, Guangdong, Guangzhou 510282, China

## Abstract

*Ethnopharmacological Relevance*. The Hugan Qingzhi tablet (HQT) is a traditional Chinese medicine used for treating NAFLD (nonalcoholic fatty liver disease). The present study evaluated the anti-inflammatory effects of HQT in rats with NAFLD. *Materials and Methods*. HQT was administered daily to the NAFLD experimental groups. Biochemical markers, histopathological data, and oxidative stress/antioxidant biomarkers were determined. Proinflammatory cytokines interleukin-1*β* (IL-1*β*), tumor necrosis factor *α* (TNF-*α*), and interleukin-6 (IL-6) were detected by enzyme-linked immunoassay. Expressions of silent information regulator 1 (SIRT1) and acetylated-nuclear-factor kappaB-p65 (Ac-NF-*κ*B-p65) were performed by western blotting. *Results*. At high and moderate doses, HQT was highly effective in decreasing serum alanine aminotransferase (*P* < 0.01), aspartate aminotransferase (*P* < 0.01), hepatic total cholesterol (*P* < 0.01), triglycerides (*P* < 0.01), and free fatty acid levels (*P* < 0.01). Moreover, high and moderate doses of HQT reduced hepatic levels of the proinflammatory cytokines TNF-*α* (*P* < 0.01), IL-1*β* (*P* < 0.01), and IL-6 (*P* < 0.01), enhanced SIRT1 expression, and depressed Ac-NF-*κ*B-p65 expression at protein level. *Conclusions*. In our NAFLD rat model, HQT exerted substantial anti-inflammatory and antioxidant activities, possibly involving the regulation of SIRT1 and Ac-NF-*κ*B-p65 expression.

## 1. Introduction

Nonalcoholic fatty liver disease (NAFLD) is one of the most prevalent chronic diseases in the world [1]. Histologically, NAFLD encompasses a disease spectrum ranging from simple steatosis to nonalcoholic steatohepatitis (NASH), cirrhosis, and, in some cases, hepatocellular carcinoma [2]. NAFLD is strongly linked with insulin resistance and is currently considered the hepatic manifestation of metabolic syndrome [3]. In the western countries, NAFLD prevalence ranges within 17%~33% and may reach 80% in obese people [4]. The pathogenesis of NAFLD is complex, influenced by both the expression of the host genes and environmental factors. Fat accumulation, insulin resistance, and inflammation likely play important roles in NAFLD initiation and development [5–11].

Caloric restriction is known to improve blood glucose levels and to lower blood pressure and cholesterol content [12]. Deng et al. reported that caloric restriction was beneficial for NAFLD and increased the expression of silent information regulator 1 (SIRT1) in rats fed a high-fat diet (HFD), suggesting that SIRT1 expression may be important in NAFLD [13]. SIRT1 is a SIR2 protein member, which consists of nicotinamide adenine dinucleotide-dependent histone or nonhistone deacetylases and adenosine diphosphate ribosyl transferases [14]. It is well documented that SIRT1 ameliorates NAFLD by inhibiting adiposity and inflammation [15–17]. Picard et al. found that SIRT1 upregulation triggers lipolysis and fat loss in differentiated fat cells [18]. In addition, numerous recent data have ascertained that SIRT1 inhibits nuclear factor-*κ*B (NF-*κ*B) signaling, which is closely related to tumor necrosis factor *α* (TNF-*α*), interleukin-6 (IL-6), and interleukin-1*β* (IL-1*β*). SIRT1 activation counteracts a multitude of NF-*κ*B-generated inflammatory and metabolic disturbances [19–21]. These data strongly suggest that NAFLD treatment could benefit from SIRT1 activators.

Traditional Chinese medicine (TCM) has been used to treat liver disease in China since ancient times. The* Yellow Emperor's Internal Classic*, an old manuscript containing TCM records, states that TCM has been used to treat liver diseases in China since 475 BC at least [22]. Due to its good anti-inflammatory effect and few side effects in NAFLD patients, the* Hugan Qingzhi* tablet (HQT) is an empirical formula used for ameliorating NAFLD in long-term clinical practice. HQT contains five Chinese herbal medicines: Rhizoma Alismatis, Fructus Crataegi Pinnatifidae, Folium Nelumbinis, Pollen Typhae, and Radix Notoginseng (Table 1). It has been reported that the primary components of HQT, that is, Fructus Crataegi [23] and Pollen Typhae [24], could decrease inflammation. Our previous experiments reported that HQT-treated serum had an antioxidative stress effect against NAFLD in L02 and HepG2 cells [25]. Previous preliminary findings [26] also showed that HQT treatment reduced inflammation in the liver and protected against oxidative stress in NAFLD rats. However, the detailed mechanisms involved in the anti-inflammatory action of HQT require further investigation.

Based on the critical roles of SIRT1 and NF-*κ*B in regulating lipid metabolism and inflammation and the potential capacity of HQT to prevent and control NAFLD, we hypothesized that HQT ameliorates NAFLD through the SIRT1 and NF-*κ*B signaling pathways. Therefore, the present study focused on HQT modulation of SIRT1, NF-*κ*B, TNF-*α*, IL-6, and IL-1*β* relative protein expression in a NAFLD rat model. Our results provide new mechanistic insights on how HQT exerts a beneficial effect in NAFLD.

## 2. Methods

### 2.1. Plant Material and Preparation of HQT

HQT (NO: 20101012) consists of five herbs: 6 g of Rhizoma Alismatis; 6 g of Fructus Crataegi; 3 g of Pollen Typhae; 4 g of Folium Nelumbinis; and 1 g of Radix Notoginseng (Table 1). HQT was prepared as follows: about 127 kg of a 4-herb mixture (Rhizoma Alismatis, Fructus Crataegi, Pollen Typhae, and Folium Nelumbinis) was boiled and refluxed in 720 L 70% ethanol for 2 h at 100°C, and the ethanol extract was collected, filtered, and extracted twice with the method described above. The final yield from the original dried mixture was 14.45% (w/w). Then, about 6.68 kg Radix Notoginseng was ground, sifted, and added to the dried extract to produce HQT. We have reported the methods for carefully identifying the herbs of HQT in a previous study [25]. In addition, Zhou et al. upgraded the quality standard of HQT by detecting the Fructus Crataegi content (the principal component of HQT) using thin-layer chromatography and high-performance liquid chromatography (HPLC) [27].

### 2.2. Quantitative HPLC Analysis of HQT

Standard solutions of quercetin, isorhamnetin, and 23-*O*-acetylalisol B (200 mg/mL) were combined in acetonitrile and stored at 4°C. All standard solutions were purchased from the National Institute for the Control of Pharmaceutical and Biology Products (Beijing, China). Quantitative HPLC analysis was performed using a chromatographic device (LC-20A; Shimadzu, Kyoto, Japan). The chromatographic column was a ZORBAX Eclipse Plus-C18 column (4.6 mm, 150 mm, particle size 5 *μ*m; Agilent Technologies, Santa Clara, CA, USA) used at 30°C. The mobile phase conditions were acetonitrile (A) and 0.005% formic acid in water (B). The gradient flow was as follows: 0~40 min, 85~12% B; 40~60 min, 12% B. The flow rate was 0.8 mL/min; room temperature was set at 30°C. Ultraviolet detection was performed at 210 nm.

### 2.3. Animals and Experimental Design

Normal male Sprague-Dawley rats weighing 180~220 g were supplied by the Southern Medical University of China Experimental Animal Centre (animal qualified number: 0099716; Guangzhou, China). Normal diet was purchased from the same center. The HFD component parts are presented in Table 2. The HFD was acquired from the Guangdong Province Medicine Experimental Animal Centre, China. All animal experimentation and maintenance protocols were approved by the Southern Medical University Animal Ethics Committee and carried out in accordance with the institutional guidelines. The rats were kept on a regular 12 h light/dark circle in a 25 ± 2°C and humidity-controlled vivarium. After 1-week adaptation, rats were divided into six groups comprising initially 10 rats each: control (Con, normal diet, distilled water), HFD (HF, 1 mL/100 g body weight [BW], distilled water), fenofibrate (FF, 0.1 g/kg BW fenofibrate suspension [28]), and HQT high/moderate/low dosage (HH/HM/HL, 2.16/1.08/0.54 g/kg BW HQT suspension); the corresponding treatments were administered to each group daily. All but the Con group were fed HFD for 12 weeks to induce NAFLD. Water and the diets were available* ad libitum* and BW was recorded weekly.

### 2.4. Biochemical Analysis

Following the 12-week treatment period, the rats were fasted overnight before being euthanized by anesthesia with chloral hydrate (100 mg/kg BW). Then, serum samples from each group of rats were collected, centrifuged with 3000 rpm for 10 min at 4°C, and stored at −80°C. The liver weight of each rat was recorded and the hepatic index calculated as follows: liver weight/body weight × 100%. Serum lipid profile and liver function tests (aspartate aminotransferase (AST), alanine aminotransferase (ALT)) were assayed using an Olympus AU5400 biochemical analyzer (Tokyo, Japan) in Nanfang Hospital (Guangzhou, China).

### 2.5. Hepatic Histology

After the animals were euthanized, the hepatic tissues were weighed, minced, immediately frozen in liquid nitrogen, and stored at −80°C prior to subsequent analyses. Four to five liver samples were randomly selected from each group and fixed in 10% formalin for histological examination. Then, the paraffin-embedded tissues were stained with hematoxylin and eosin (H&E). Total lipids were extracted from the hepatic tissues by oil red O staining. Oil red O and the H&E staining reagents were obtained from Sigma-Aldrich (St. Louis, MO, USA). NAFLD activity scores were used to assess changes in the histological features of each group following H&E staining [29]. Three histological features were adopted and evaluated semiquantitatively using this score: hepatic steatosis (0~3), lobular inflammation (0~2), and hepatocellular ballooning (0~2). The degree of liver steatosis was assessed by oil red O staining; the Olympus IPP6.1 image software was used to conduct the quantitative analysis and calculate the oil red O staining areas.

### 2.6. Determination of Lipid Contents in Hepatic Tissue

The hepatic tissues were homogenized in 10 mg/mL physiological saline and incubated at 4°C for 2 h. Samples were then centrifuged at 5000 ×g for 15 min, and the suspension was collected for subsequent determination of liver total cholesterol (CHOL) and triglyceride (TG) content by the colorimetric method using an Olympus AU5400 clinical biochemical analyzer at Nanfang Hospital. Free fatty acid (FFA) determination was performed with a rat FFA enzyme-linked immunosorbent assay (ELISA) kit (Yanji-Biochemical, Shanghai, China).

### 2.7. Hepatic Malondialdehyde and Antioxidant Defense Levels

Hepatic homogenates and mitochondrial fractions were used to assess antioxidant defense levels. Assay kits for glutathione peroxidase (GSH-PX), malondialdehyde (MDA), superoxide dismutase (SOD), and serum total antioxidant capacity (TAOC) were obtained from Nanjing Jiancheng (Nanjing, China). MDA was measured using the method of Ohkawa et al. [30]. The analysis was conducted according to the kit instructions.

### 2.8. Cytokine Assay

The proinflammatory cytokines TNF-*α*, IL-6, and IL-1*β* in the hepatic homogenates were measured using commercial ELISA (Sunny ELISA Kits; Mutisciences, Hangzhou, China) according to the kit user guide.

### 2.9. Western Blot Analysis

Total protein extracts were obtained by lysing frozen hepatic tissue (100 mg) using the Total Protein Extraction Kit (Beyotime Biotech). Protein concentrations were quantified by the BCA Protein Concentration Determination Kit (Beyotime Biotech). Proteins (40 *μ*g) were separated by 10% sodium dodecyl sulphate (SDS) polyacrylamide gel electrophoresis and transferred onto nitrocellulose membranes (PALL). The membranes were blocked with 5% nonfat dry milk in TBST for 1 h at room temperature and then incubated at 4°C overnight with the corresponding primary antibodies (SIRT1, 1 : 1000, CST; Ac-NF-*κ*B-p65, 1 : 200, CST; GAPDH, 1 : 1000, Beyotime). Then, bound antibodies onto membranes were detected by using the secondary antibody (1 : 10000). Subsequently, enhanced ECL reagent was used to visualize the membranes. Membranes were exposed and the band intensities were analyzed by Gel-Pro Analyzer 4.0 (Media Cybernetics, Rockville, MD, USA). The results are expressed as the ratio of SIRT1 and Ac-NF-*κ*B-p65 to GAPDH densitometry.

### 2.10. Statistical Analysis

All results were expressed as means ± SD, and the one-way analysis of variance was used to analyze the mean values in the different groups, followed by a post hoc Dunnett's or Bonferroni's multiple comparisons test. Statistical significance corresponded to *P* < 0.05.

## 3. Results

### 3.1. HPLC Analysis of HQT

**Figure 1:**
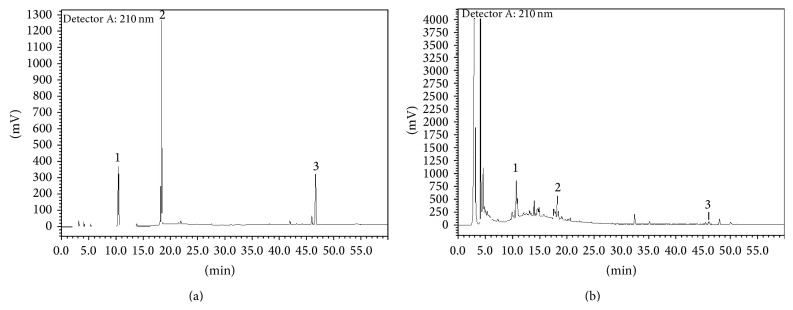
HPLC pattern of HQT. (a) Standard reference material. Isorhamnetin glycoside (10.632 min, peak 1), quercetin (18.412 min, peak 2), and 23-*O*-acetylalisol B (46.5 min, peak 3). (b) HQT.

HPLC analyses of HQT and the standards are presented in Figures 1(a) and 1(b). The curves showed that HQT contained complex components. The component retention times were 10.6 min (isorhamnetin glycoside), 18.4 min (quercetin), and 46.5 min (23-*O*-acetylalisol B).

### 3.2. General Evaluation, BW, and Hepatic Index of Rats

**Figure 2:**
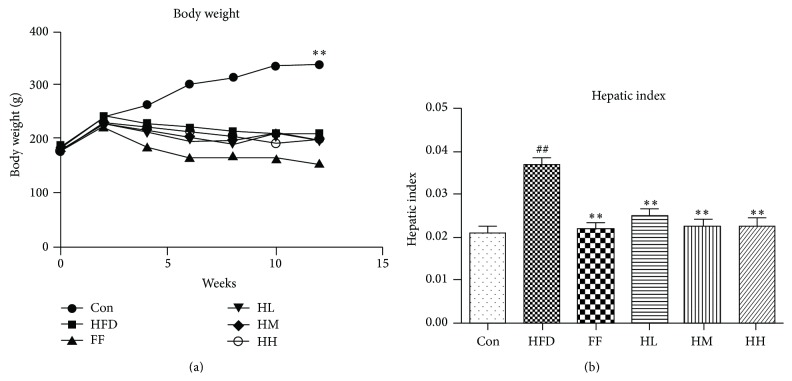
Rat (a) BW and (b) hepatic index changes (mean ± SD). Hepatic index = liver weight/body weight × 100%. Con: control group (*n* = 10); HFD: high-fat diet group (*n* = 8); FF: HFD + fenofibrate group (*n* = 7); HL: HFD + low-dose HQT group (*n* = 9); HM: HFD + moderate-dose HQT group (*n* = 10); HH: HFD + high-dose HQT group (*n* = 10). ^#^ *P* < 0.05, ^##^ *P* < 0.01 versus Con group. ^*∗*^ *P* < 0.05, ^*∗∗*^ *P* < 0.01 versus HFD group.

Con group rats had regular diet and development. During modeling, HFD group rats ate less, their fur tended to be yellow, and they gained weight and maintained total active movement. The BW in all groups increased (Figure 2(a)). The hepatic index was significantly increased in the HFD group compared with the Con group (*P* < 0.01, Figure 2(b)). However, the hepatic index was significantly decreased in the HQT and FF groups (*P* < 0.01 versus the HFD group).

### 3.3. Analysis of Serum Biochemical Parameters

**Figure 3:**
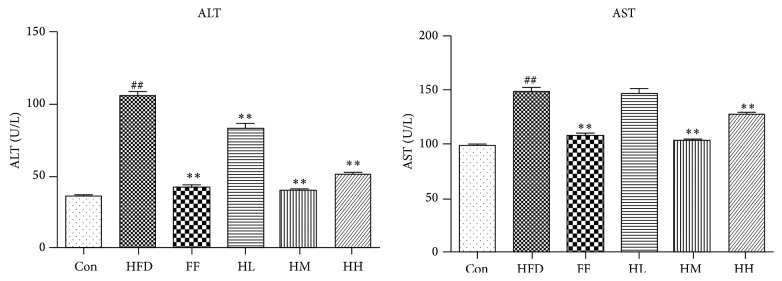
Changes in ALT and AST (mean ± SD). Con: control group (*n* = 10); HFD: high-fat diet group (*n* = 8); FF: HFD + fenofibrate group (*n* = 7); HL: HFD + low-dose HQT group (*n* = 9); HM: HFD + moderate-dose HQT group (*n* = 10); HH: HFD + high-dose HQT group (*n* = 10). ^#^ *P* < 0.05, ^##^ *P* < 0.01 versus Con group. ^*∗*^ *P* < 0.05, ^*∗∗*^ *P* < 0.01 versus HFD group.

HQT administration notably attenuated the increased levels of AST (*P* < 0.01 versus HFD group, including HL/HM/HH groups) and ALT (*P* < 0.01 versus HFD group, including HM/HH groups) caused by the long-term HFD (Figure 3). In addition, AST and ALT activities in the FF group were decreased.

### 3.4. Analysis of Hepatic Histopathology

**Figure 4:**
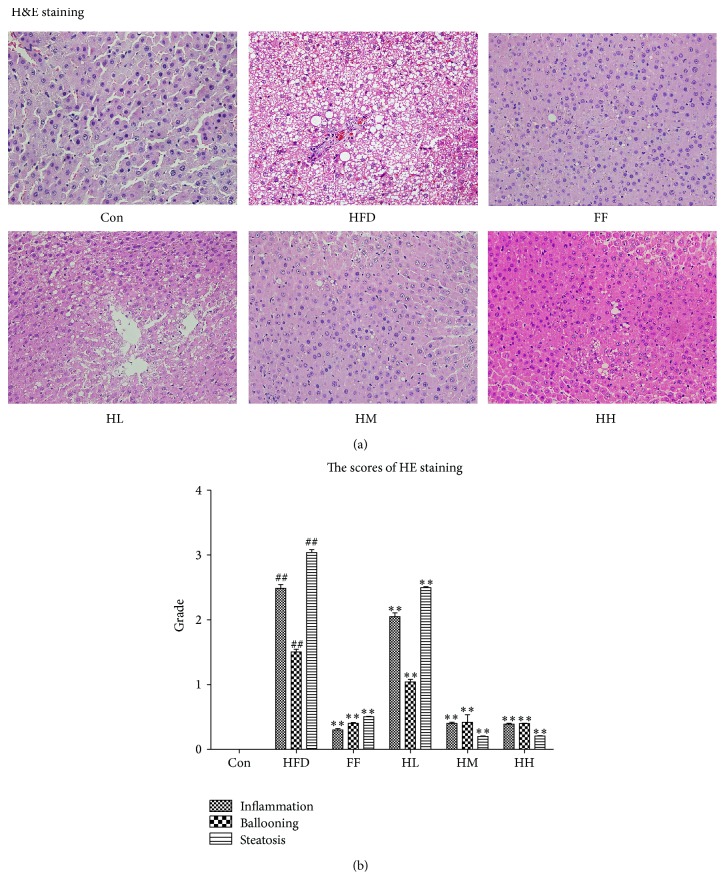
H&E-stained liver tissue in each group (magnification ×400). Con: control group; HFD: high-fat diet group; FF: HFD + fenofibrate group; HL: HFD + low-dose HQT group; HM: HFD + moderate-dose HQT group; HH: HFD + high-dose HQT group.

**Figure 5:**
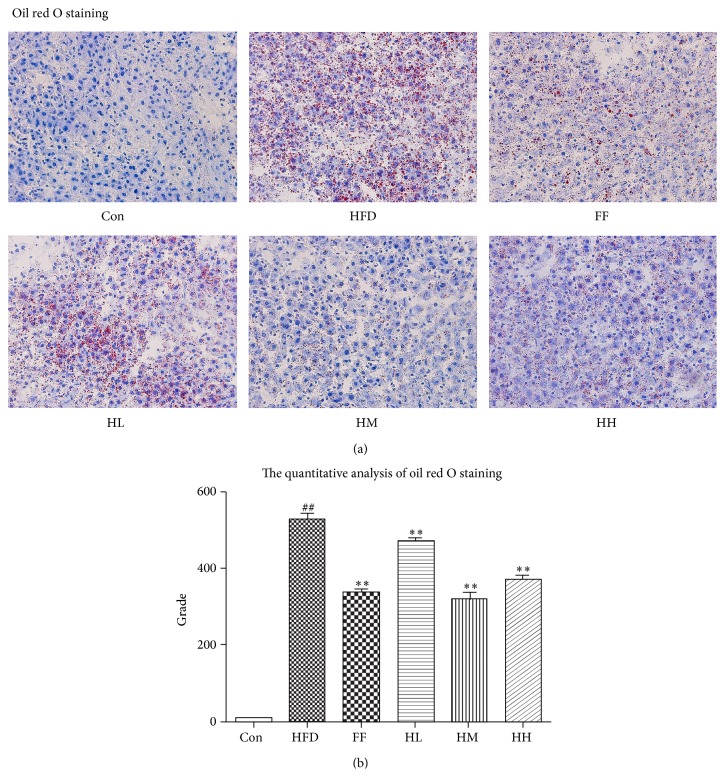
Oil red O-stained liver tissue of each group (magnification ×400). Con: control group; HFD: high-fat diet group; FF: HFD + fenofibrate group; HL: HFD + low-dose HQT group; HM: HFD + moderate-dose HQT group; HH: HFD + high-dose HQT group.

Photomicrographs of H&E-stained hepatic sections are shown in Figure 4(a). The histological category scores for each group are presented in Table 3 and Figure 4(b) [29]. No fatty infiltration was observed in the Con group. Rats in the HFD group developed macrovesicular steatosis, steatohepatitis changes, inflammation, and massive infiltration of inflammatory cells around the central vein. By contrast, HQT ameliorated these morphological features in the HH, M, and FF groups. Moreover, compared with the HFD group, hepatocyte lipid accumulation, especially in the HM, HH, and FF groups, was significantly decreased (Figures 5(a) and 5(b)).

### 3.5. Evaluation of Lipid Contents in Hepatic Tissue

**Figure 6:**
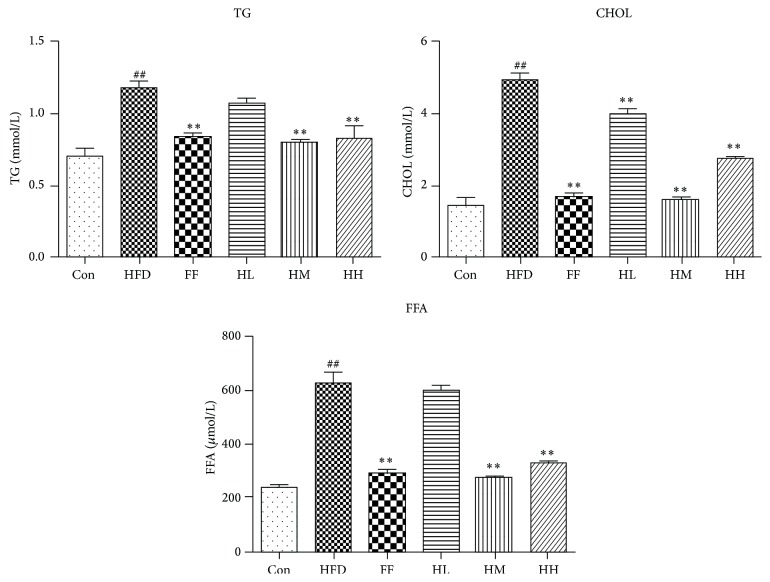
Changes in TG, CHOL, and FFA levels of liver homogenates (mean ± SD). Con: control group (*n* = 10); HFD: high-fat diet group (*n* = 8); FF: HFD + fenofibrate group (*n* = 7); HL: HFD + low-dose HQT group (*n* = 9); HM: HFD + moderate-dose HQT group (*n* = 10); HH: HFD + high-dose HQT group (*n* = 10). ^#^ *P* < 0.05, ^##^ *P* < 0.01 versus Con group. ^*∗*^ *P* < 0.05, ^*∗∗*^ *P* < 0.01 versus HFD group.

The FFA, TG, and CHOL levels in the HFD group were significantly increased compared to the Con group (*P* < 0.01, Figure 6). Compared with the HFD group, there was a notable decline in FFA, TG, and CHOL levels of the HQT (HM, HH) and FF groups (*P* < 0.01).

### 3.6. Evaluation of Antioxidant Defense Levels of Hepatic Tissue

**Figure 7:**
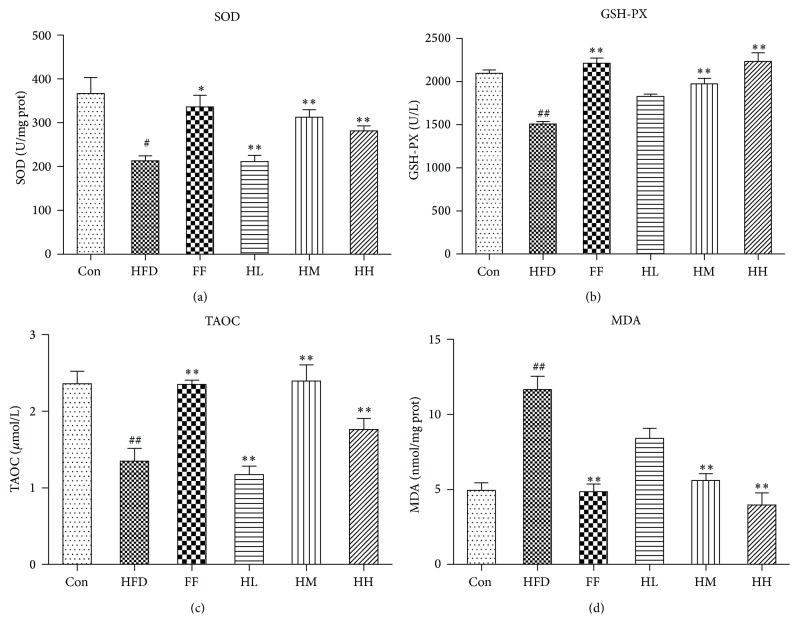
Changes in (a) SOD, (b) GSH-PX, (c) TAOC, and (d) MDA levels of each group (mean ± SD). Con: control group (*n* = 10); HFD: high-fat diet group (*n* = 8); FF: HFD + fenofibrate group (*n* = 7); HL: HFD + low-dose HQT group (*n* = 9); HM: HFD + moderate-dose HQT group (*n* = 10); HH: HFD + high-dose HQT group (*n* = 10). ^#^ *P* < 0.05, ^##^ *P* < 0.01 versus Con group. ^*∗*^ *P* < 0.05, ^*∗∗*^ *P* < 0.01 versus HFD group.

Compared to the Con group, the HFD group had significantly increased MDA levels (Figure 7(d)) (*P* < 0.01), which contrasted with the significantly decreased SOD, GSH-PX, and TAOC levels (*P* < 0.05 or *P* < 0.01). In comparison with the HFD group, the HM, HH, and FF groups had reduced MDA levels (*P* < 0.01) and increased GSH-PX (*P* < 0.01), SOD, and TAOC levels (*P* < 0.01).

### 3.7. Determination of Cytokine Levels in Hepatic Tissue

**Figure 8:**
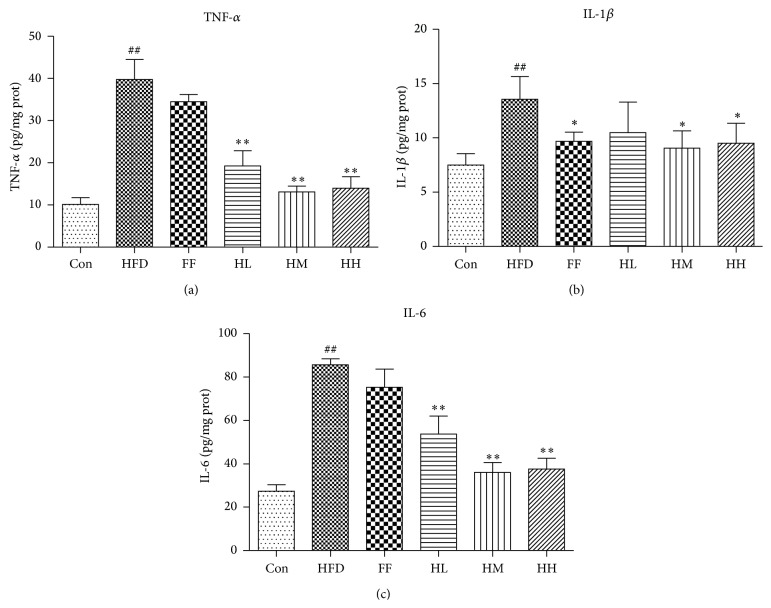
Liver (a) TNF-*α*, (b) IL-1*β*, and (c) IL-6 expression of each group (mean ± SD). Con: control group (*n* = 10); M: high-fat diet group (*n* = 8); FF: HFD + fenofibrate group (*n* = 7); HL: HFD + low-dose HQT group (*n* = 9); HM: HFD + moderate-dose HQT group (*n* = 10); HH: HFD + high-dose HQT group (*n* = 10). ^#^ *P* < 0.05, ^##^ *P* < 0.01 versus Con group. ^*∗*^ *P* < 0.05, ^*∗∗*^ *P* < 0.01 versus HFD group.

Compared with the Con group (Figure 8), IL-6, IL-1*β*, and TNF-*α* levels in the HFD group were drastically elevated (*P* < 0.01). IL-6 and TNF-*α* levels in the HQT groups (HL/HM/HH) were significantly decreased compared to the HFD group (*P* < 0.01). However, IL-6 and TNF-*α* levels in the FF group were not statistically decreased as compared to the HFD group. The HM and HH groups had decreased IL-1*β* levels compared to the HFD group (*P* < 0.05). Of the three HQT groups, the HM group exerted the best effect for decreasing the inflammatory factors. The FF group had significantly reduced IL-1*β* levels (*P* < 0.05) compared with the HFD group.

### 3.8. Hepatic SIRT1 and Ac-NF-*κ*B-p65 Expression

**Figure 9:**
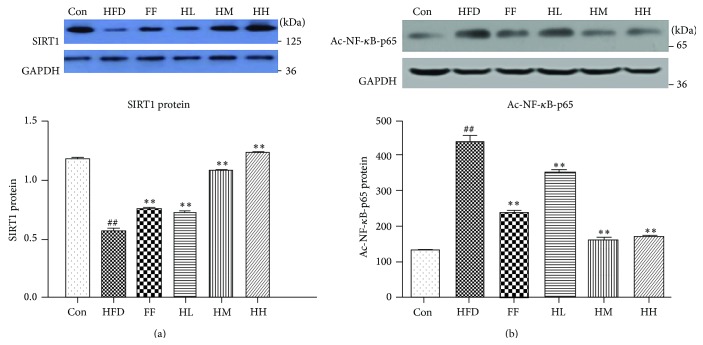
Liver (a) SIRT1 and (b) Ac-NF-*κ*B-p65 expression of each group (mean ± SD). Con: control group; M: high-fat diet group; FF: HFD + fenofibrate group; HL: HFD + low-dose HQT group; HM: HFD + moderate-dose HQT group; HH: HFD + high-dose HQT group. ^#^ *P* < 0.05, ^##^ *P* < 0.01 versus Con group. ^*∗*^ *P* < 0.05, ^*∗∗*^ *P* < 0.01 versus HFD group.

SIRT1 expression in the HFD group was significantly decreased (*P* < 0.05, Figure 9(a)) compared with the Con group. In contrast, SIRT1 expression was markedly elevated in the HQT (HL/HM/HH) and FF groups (*P* < 0.01) compared with the HFD group. In the HFD group, hepatic Ac-NF-*κ*B-p65 expression was significantly increased as compared with the Con group (*P* < 0.01, Figure 9(b)). Compared with the HFD group, the HH/HM and FF groups had significantly decreased Ac-NF-*κ*B-p65 expression (*P* < 0.01).

## 4. Discussion

Over the past decade, NAFLD has become one of the most prevalent causes of chronic liver disease, affecting both adults and children [31]. It is believed that approximately 10%~20% of NAFLD patients develop NASH [32]. Low-grade chronic inflammation is widely recognized as a salient feature of NAFLD and of many of its accompanying disorders [33]. HQT has been identified as a potential modulator of NAFLD through its lipid-lowering and anti-inflammatory effects [25, 26]. However, the mechanisms whereby HQT exerts its anti-inflammatory effects in NAFLD rats remain to be elucidated. This study aimed to verify the ability of HQT to modulate SIRT1, IL-6, IL-1*β*, TNF-*α*, and Ac-NF-*κ*B-p65 expression, which is closely related to inflammation in NAFLD rats.

We established a rat model of NAFLD by using a HFD over a 12-week period [34]. Hepatic histological data as well as serum biochemical markers and oxidative stress indicators confirmed the validity of the model. High and moderate concentrations of HQT (2.16/1.08 g/kg BW) were sufficient for reducing the accumulation of lipids such as TG, CHOL, and FFA. This result and the hepatic lipid-lowering action of HQT observed in FFA-induced L02 and HepG2 cells strongly highlight the antisteatosis and antioxidant potential of HQT [25]. Oxidative stress could be triggered by FFA due to increased mitochondrial uncoupling [35, 36] and oxidation [37, 38], leading to increased inflammation. We find it noteworthy that, compared with the HFD group, the high and moderate concentrations of HQT decreased oxidative stress injury indicators such as MDA levels, with a concomitant increase in SOD, GSH-PX, and TAOC levels. Moreover, HQT decreased the HFD-induced elevation of serum ALT and AST significantly, indicating its beneficial effect on hepatocyte injury. Cohen et al. found that long-term HFD intake damaged hepatic architecture and produced histological changes such as microvesicular and macrovesicular lipid accumulation, inflammatory infiltration, hepatocyte ballooning, and cell death in the liver [39]. Treatment with high and moderate concentrations of HQT decreased effectively steatosis and inflammation, indicating that HQT was effective for alleviating NAFLD progression.

SIRT1 is one of the mammalian sirtuin members corresponding to class III histone deacetylases that regulate senescence, stress resistance, metabolism, and inflammation [40]. In particular, SIRT1 is potentially a pivotal molecule in the modulation of inflammation for treating NAFLD [33]. NF-*κ*B is a nuclear transcription factor widely present in many cells which regulates a variety of cytokines involved in inflammation, adhesion molecules, and protease gene transcription* in vivo* [41]. SIRT1 deacetylates p65 and interferes with the NF-*κ*B signaling pathway, thereby acting as an anti-inflammatory factor on NAFLD [42–44]. Similarly, previous studies have demonstrated that knockdown of SIRT1 can result in enhanced activation of LPS-stimulated NF-*κ*B and expression of proinflammatory cytokines such as TNF-*α*, IL-1*β*, and IL-6 [45]. Cao et al. demonstrated that amyloid beta- (A*β*-) induced IL-8 and IL-6 expression was attenuated in cells pretreated with SIRT1 activators and that SIRT1 knockdown exacerbated the A*β*-induced proinflammatory effects [46]. Recently, several studies have shown that fenofibrate exerted protective effects against TNF-*α*-induced CD40 expression through SIRT1-mediated deacetylation of the NF-*κ*B-p65 subunit [47]. Similarly, we have found that fenofibrate and HQT (HM/HH groups) increased SIRT1 and decreased Ac-NF-*κ*B-p65, TNF-*α*, IL-1*β*, and IL-6, which suggests that the beneficial effect of HQT on anti-inflammation might be partly attributable to the upregulated expression of SIRT1. SIRT1 activators, such as resveratrol, silibinin, and quercetin, have antioxidant and anti-inflammatory effects by modulating NF-*κ*B and mitogen-activated protein kinase- (MAPK-) dependent signaling pathways [48–50]. In accordance with this, another study found that quercetin significantly attenuated inflammation in NAFLD rats by increasing SIRT1 expression [51]. HPLC shows that quercetin is one of the main active components of HQT, which supports the notion that HQT possesses anti-inflammation activity. Numerous studies have indicated that long-term HFD intake leads to FFA accumulation in rat liver [52]. However, the FFA accumulation that elicits a number of damaging effects, termed lipotoxicity, also induces the NF-*κ*B activation leading to inflammation [53, 54]. Rodgers and Puigserver showed that adenoviral knockdown of SIRT1 reduced the expression of fatty acid *β*-oxidation genes in the livers of fasted mice [55]. Resveratrol, a SIRT1 activator, reported to have lipotoxicity-preventive activity, depends on the regulation of the AMPK/SIRT1/PGC1*α* (PPAR-*γ* coactivator 1*α*) axis [56]. In this study, high and moderate concentrations of HQT significantly reduced TG, CHOL, and FFA levels compared to HFD group, which further supports the idea that liver lipotoxicity was relieved. Based on this result, we propose that the anti-inflammatory effect of HQT might occur through indirect enhancement of SIRT1 expression. Further studies are needed to elucidate the precise underlying mechanism.

Quercetin, isorhamnetin, and 23-*O*-acetylalisol B are major constituents of HQT. Quercetin is one of the main effective components in Fructus Crataegi; quercetin treatment attenuated most symptoms of metabolic syndrome in a rat model of diet-induced metabolic syndrome, including abdominal obesity, cardiovascular remodeling, and NAFLD, the most likely mechanism being decreased oxidative stress and inflammation [57]. Dong et al. suggested that quercetin may inactivate NF-*κ*B expression by upregulating SIRT1 expression to ameliorate hepatocyte inflammation [58]. Moreover, isorhamnetin prevents acute inflammation by blocking NF-*κ*B activation [59]. Rhizoma Alismatis, whose main component is 23-*O*-acetylalisol B, is also helpful in preventing oxidative stress by reducing lipid peroxidation and activating antioxidant enzymes [60]. Our results provide evidence that HQT could positively modulate SIRT1 and decrease Ac-NF-*κ*B-p65 and several inflammatory cytokines, such as TNF-a, IL-6, and IL-1*β*. These results suggest that HQT has a beneficial anti-inflammatory activity against tissue damage. It is possible that HQT modulation protein's pathways are interconnecting. More researches are necessary to better understand this relationship and to provide data for using HQT as a future treatment in NAFLD.

## 5. Conclusions

HQT treatment of NAFLD is highly effective for regulating oxidative stress and decreasing liver inflammation. Further, this beneficial effect of HQT may be associated with increased hepatocytes SIRT1 and decreased Ac-NF-*κ*B-p65. Our findings suggest that HQT is a promising candidate for NAFLD prevention and control.

## Figures and Tables

**Table 1:** Herbal constituents of *Hugan Qingzhi*.

Pharmaceutical name	English name	Botanical name	Family	Part used	Chinese name	Ratio
Rhizoma Alismatis	Rhizome of oriental water plantain	*Alisma orientalis* (Sam.) Juzep.	Alismataceae	Rhizome	*Ze Xie *	6
Fructus Crataegi	Hawthorn fruit	*Crataegus pinnatifida* Bunge	Rosaceae	Fruit	*Shan Zha *	6
Pollen Typhae	Cattail pollen	*Typha orientalis* C. Presl	Typhaceae	Pollen	*Pu Huang *	3
Folium Nelumbinis	Lotus leaf	*Nelumbo nucifera* (Gaertn.)	Nymphaeaceae	Leaf	*He Ye *	4
Radix Notoginseng	Sanchi	*Panax pseudoginseng* var. notoginseng	Araliaceae	Root	*San Qi *	1

**Table 2:** HFD and calories contained.

Ingredient	HFD (g/kg)	Calories (kJ/kg)
Ordinary mouse feed (74.3%)	743	12408.1
Lard (10%)	100	346.0168
Soya oil (3%)	30	112.84248
Cane sugar (10%)	100	162.7576
Cholesterol (2%)	20	—
Sodium cholate (0.5%)	5	—
Propylthiouracil (0.2%)	2	—
Total		13029.72

**Table 3:** Histological variable scoring following H&E staining.

Group	Inflammation (0~3)	Ballooning (0~2)	Steatosis (0~3)
Con	0	0	0
HFD	2.5	1.5	3
FF	0.3	0.2	0.5
HL	2	1	2.5
HM	0.4	0.3	0.2
HH	0.4	0.4	0.2

Con: control; HFD: high-fat diet; FF: HFD + fenofibrate; HL: HFD + low-dose HQT; HM: HFD + moderate-dose HQT; HH: HFD + high-dose HQT.

## Abbreviations

ALT: Alanine aminotransferase
AST: Aspartate aminotransferase
FF: Fenofibrate
FFA: Free fatty acid
HFD: High food diet
HPLC: High-performance liquid chromatography
HQT: Hugan Qingzhi tablet
IL-1*β*: Interleukin-1*β*
IL-6: Interleukin-6
MDA: Malondialdehyde
NAFLD: Nonalcoholic fatty liver disease
NASH: Nonalcoholic steatohepatitis
NF-*κ*B: Nuclear-factor kappaB
Ac-NF-*κ*B-p65: Acetylated-nuclear-factor kappaB-p65
PPAR*α*: Peroxisome proliferator activated receptor-*α*
SIRT1: Silent information regulator 1
SOD: Superoxide dismutase
T-AOC: Total antioxidant capacity
TG: Triglyceride
TNF-*α*: Tumor necrosis factor *α*.

## Chemical Constituents Studied in This Paper

Quercetin (PubChem CID: 5280343)
23-*O*-Acetylalisol B (PubChem CID: 3084460)
Ursolic acid (PubChem CID: 64945)
Nuciferine (PubChem CID: 10146)
Isorhamnetin-3-*O*-neohesperidoside (PubChem CID: 24204448).
